# Multimodal artificial intelligence in epilepsy: antiseizure medication response, epileptogenic zone localization, and surgical outcome prediction

**DOI:** 10.3389/fneur.2026.1957037

**Published:** 2026-09-22

**Authors:** Lin Yang, Hanyue Wang, Zuhong Lu, Yu Sun

**Affiliations:** 1 School of Biological Science and Medical Engineering, Southeast University, Nanjing, China; 2 Department of Radiology, The First Affiliated Hospital of Soochow University, Suzhou, China; 3 School of Public Health, Suzhou Medical College of Soochow University, Suzhou, China; 4 International Lab for Child Medical Imaging Research, School of Biological Science and Medical Engineering, Southeast University, Nanjing, China; 5 Institute of Cancer and Genomic Sciences, University of Birmingham, Birmingham, United Kingdom

**Keywords:** antiseizure medication response, deep learning, electroencephalography, epilepsy, epileptogenic zone localization, multimodal artificial intelligence, neuroimaging, surgical outcome prediction

## Abstract

Epilepsy remains difficult to manage because approximately 30% of patients develop drug-resistant epilepsy, epileptogenic zones may remain occult on conventional magnetic resonance imaging, and surgical outcomes vary substantially. Multimodal artificial intelligence (AI) can integrate structural and functional MRI, positron emission tomography, diffusion imaging, electroencephalography, intracranial electrophysiology, and clinical information to support quantitative decision-making. This targeted narrative review synthesizes published evidence on three clinical tasks: prediction of antiseizure medication response, localization of epileptogenic zones, and prediction of surgical outcomes. We summarize machine-learning, deep-learning, and brain-network approaches, together with representative validation strategies and performance metrics. Current studies demonstrate promising task-specific performance, but their results should not be compared directly because cohorts, endpoints, modalities, and validation designs differ markedly. Major barriers include small single-center datasets, inconsistent acquisition and annotation, incomplete multimodal data, limited external validation, and insufficient interpretability. Future priorities include standardized multicenter datasets, prospective evaluation, privacy-preserving collaboration, interpretable fusion models, and clinically integrated human-AI workflows. At present, multimodal AI should be regarded as supervised decision support rather than a replacement for specialist interpretation.

## 1 Introduction

Epilepsy affects over 70 million individuals worldwide, imposing a heavy socioeconomic burden due to recurrent seizures, cognitive comorbidities, and premature mortality (1). Despite the availability of more than 20 antiseizure medications (ASMs), drug-resistant epilepsy (DRE) develops in 20–30% of patients, who fail to achieve seizure freedom with adequate trials of two tolerated ASMs (2). For DRE patients, surgical resection or neuromodulation is the mainstay treatment, but success depends critically on precise localization of the epileptogenic zone (EZ). However, 15–30% of focal epilepsy patients present with MRI-negative findings on conventional clinical sequences, making EZ localization extremely difficult (3). Moreover, postoperative seizure freedom rates vary widely (30–70%) across centers, lacking reliable preoperative predictors for individualized surgical decision-making (4).

Traditional neuroimaging analysis depends on qualitative visual assessment, which is subjective, time-consuming, and insensitive to subtle structural or functional abnormalities (5). The emergence of artificial intelligence (AI), particularly machine learning (ML), deep learning (DL), and graph-based brain network analysis, enables automated extraction of high-dimensional quantitative features from multimodal data, supporting quantitative diagnostic and predictive analysis (6). Multimodal fusion of structural MRI, PET-CT, DTI, rs-fMRI, and EEG compensates for single-modality limitations, providing complementary anatomical, metabolic, microstructural, functional, and electrophysiological information (7). (Table 1). This review focuses on DL-dominated AI applications in multimodal epilepsy imaging, covering three core clinical pain points: ASM response prediction, EZ localization in MRI-negative epilepsy, and surgical outcome evaluation. We analyze technical evolution, model performance, current limitations, and mitigation strategies for small-sample constraints. We also outline future research priorities and the transformative value of AI in individualized epilepsy precision medicine.

**Table 1:** Principal multimodal data sources in epilepsy AI and their clinical roles.

Modality (abbreviation)	Principal signal and clinical role	Key strengths and limitations
Structural MRI	Anatomical structure and lesion morphology; detects hippocampal sclerosis, focal cortical dysplasia, tumors, and other structural substrates.	High spatial resolution and no ionizing radiation; sensitivity remains limited for subtle or MRI-negative lesions and it provides limited functional or metabolic information.
18F-FDG PET/CT	Regional glucose metabolism with anatomical co-registration; identifies interictal hypo-metabolism or ictal hyper-metabolism associated with epileptogenic tissue.	Useful in MRI-negative or discordant cases; limited spatial resolution, radiation exposure, cost, and variable specificity.
Diffusion tensor imaging (DTI)	White-matter microstructure and structural connectivity through measures such as fractional anisotropy and mean diffusivity.	Complements structural MRI and supports network analysis; sensitive to motion, acquisition differences, and tractography assumptions.
Resting-state fMRI (rs-fMRI)	Spontaneous blood-oxygen-level-dependent activity and functional connectivity; characterizes distributed epileptic networks.	Whole-brain and non-invasive; lower temporal resolution than EEG and vulnerable to physiological and motion artifacts.
Electroencephalography (EEG)	Neuronal electrical activity with interictal and ictal discharges; supports seizure-onset and network localization.	Excellent temporal resolution, low cost, and broad availability; limited spatial/depth resolution and sensitivity to scalp and recording artifacts.

### 1.1 Review methodology

This targeted narrative review considered English-language literature indexed in PubMed/MEDLINE, Web of Science, Scopus, and IEEE Xplore from January 2018 through 30 June 2026, together with selected foundational studies and professional guidance. Search concepts combined epilepsy; antiseizure medication response or drug resistance; epileptogenic zone or seizure-onset zone; surgical outcome; artificial intelligence, machine learning, deep learning, or brain-network analysis; and MRI, PET, DTI, fMRI, EEG, or intracranial EEG. Peer-reviewed original studies, systematic reviews, meta-analyses, consensus documents, and published conference proceedings directly relevant to the three pre-specified clinical tasks were prioritized. Titles, abstracts, and relevant full texts were assessed for direct relevance, methodological clarity, reported validation, and clinically interpretable outcomes. Non-English reports, studies without sufficient methodological information, and duplicate reports were excluded. No systematic-review protocol or formal risk-of-bias tool was applied.

## 2 Artificial intelligence technologies in epilepsy research

AI has advanced rapidly and been widely applied in epilepsy research, evolving from traditional machine learning to deep learning and brain network analysis. These approaches have overcome limitations of conventional analytics and enabled robust quantitative study of multimodal brain imaging. This section summarizes core AI technologies, their principles, strengths, and applications (see Figure 1).

**Figure 1:**
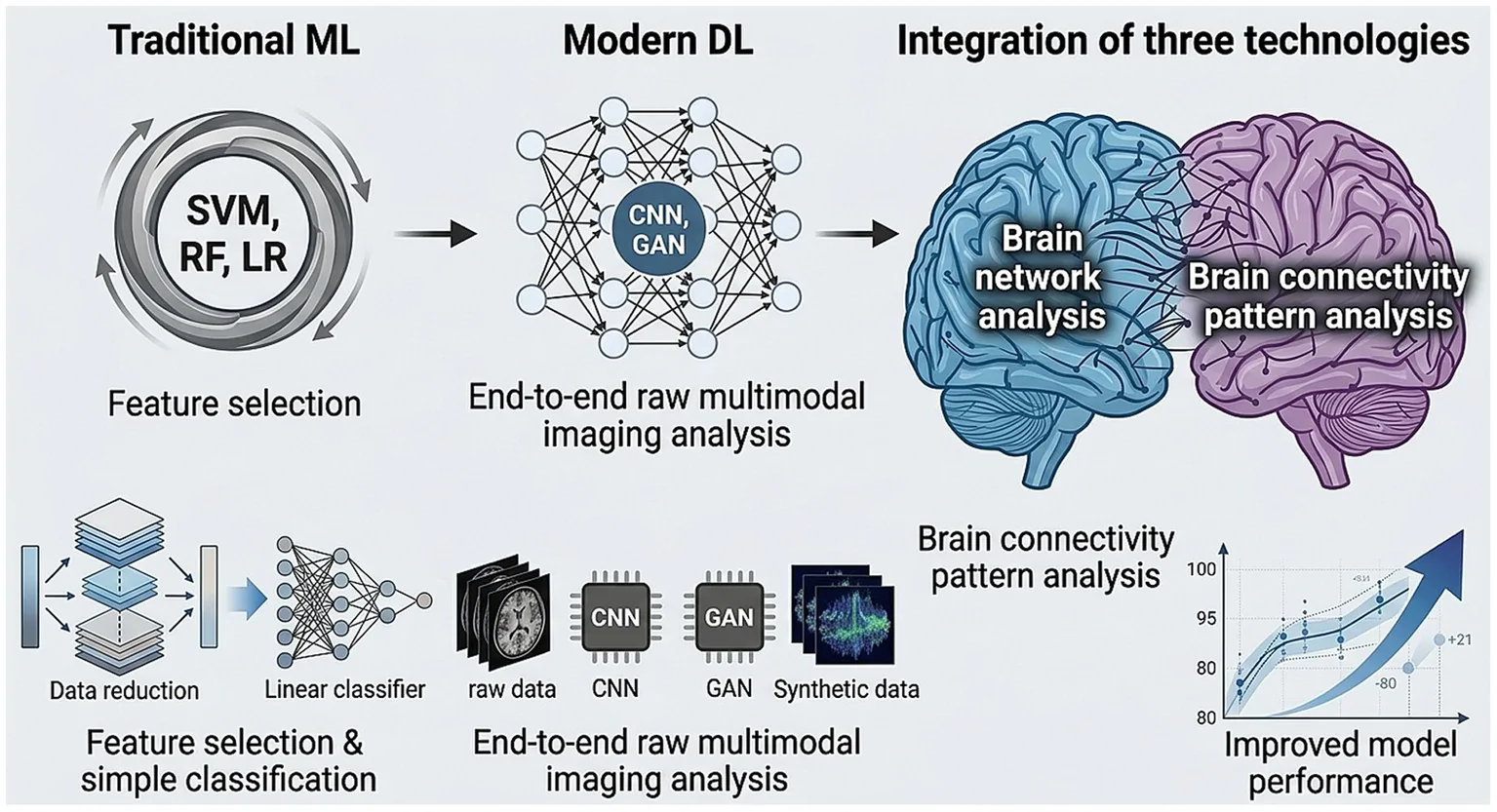
Evolution of artificial intelligence approaches in epilepsy research. Traditional machine learning relies on handcrafted feature selection and conventional classifiers, whereas deep learning enables end-to-end analysis of multimodal data. Integration with brain-network analysis further supports characterization of connectivity patterns and improves model performance.

### 2.1 Traditional machine learning

Classical machine learning methods rely on manual feature engineering, model training, and validation to support classification and prediction. They have been used for drug response prediction, epileptogenic focus localization, and surgical outcome evaluation (6). However, they suffer from heavy dependence on handcrafted features, limited ability to capture high-dimensional patterns, and poor generalization in small datasets. With increasingly complex imaging data, deep learning has become the mainstream framework.

### 2.2 Deep learning

Deep learning uses deep neural networks to automatically learn hierarchical features directly from raw data, avoiding manual feature extraction. Common models include CNN, GAN, RNN, and Transformer, supporting lesion segmentation, focus localization, multimodal fusion, and seizure prediction (6). It enables end-to-end analysis with high performance but requires large labeled datasets, lacks interpretability as a black-box system, and demands significant computational resources.

### 2.3 Brain network analysis

Based on graph theory, brain network analysis models brain regions as nodes and connections as edges, revealing system-level connectivity abnormalities in epilepsy. It provides interpretable biomarkers for diagnosis, focus localization, and treatment evaluation (6). While effective and biologically meaningful, it is sensitive to network construction parameters, faces challenges in metric selection, and remains limited in integrating imaging and electrophysiological data.

Recently, the integration of deep learning and brain network analysis has become a research hotspot. Deep learning improves automated network feature extraction, while network analysis enhances model interpretability. Their combination synergistically boosts the performance and clinical applicability of AI in epilepsy research.

## 3 AI-based prediction of antiseizure medication response in epilepsy

The therapeutic response to anti-seizure medications (ASMs) exhibits substantial interindividual variability. ASM resistance represents a multifactorial phenotype involving genetic polymorphisms, aberrant brain network connectivity, metabolic alterations, and microstructural changes (8). Conventional predictive indicators—such as seizure frequency, age at onset, and lesion type—yield relatively low accuracy (AUC < 0.70) and are insufficient to guide early individualized drug selection (9). At present, there is a lack of effective prospective evaluation tools in clinical practice; treatment decisions rely largely on evidence-based guidelines and physician experience, which often compel patients to undergo repeated, sequential drug trials. This approach not only prolongs the duration of uncontrolled seizures and increases the risks of status epilepticus, accidental injury, and sudden unexpected death in epilepsy (SUDEP), but may also—owing to persistent epileptiform discharges—induce neuronal damage and neural network remodeling, thereby accelerating cognitive decline and the development of psychiatric comorbidities. Therefore, the ability to predict a given patient’s response to different ASMs prior to the first prescription holds critical clinical importance for averting ineffective therapy, optimizing drug selection, shortening the time to seizure control, and mitigating irreversible neurological impairment.

### 3.1 Value of EEG and radiomic features

Electroencephalography (EEG)-derived functional network features have demonstrated unique value in predicting ASM response. A 2025 study (10) extracted frontotemporal network connectivity features from resting-state EEG and employed an ensemble learning model (Tree Bagger) to predict pharmacoresistance in newly diagnosed temporal lobe epilepsy (TLE) patients, achieving an accuracy of 91.5% (AUC = 0.92). This was the first study to introduce an ensemble approach in a prospective cohort, although the sample size was relatively small. Another EEG-based investigation (11) used a one-dimensional convolutional neural network (1D-CNN) combining EEG and clinical features in 101 patients, attaining a prediction accuracy of 84% (AUC = 0.81) for drug-resistant epilepsy (DRE). This method enabled automatic extraction of deep features, circumventing the reliance on manual feature engineering. Furthermore, Hwang et al. (12) applied a LightGBM model based on gamma coherence in TLE patients receiving monotherapy and achieved an AUC of 0.821 in a cohort of 48 patients. Through graph-theoretical analysis, that study revealed functional network reorganization in patients with DRE. Collectively, these investigations have uncovered patterns of functional connectivity reorganization in refractory epilepsy, providing electrophysiological evidence to support prediction.

Radiomics has also contributed significantly to the prediction of ASM efficacy. Lee et al. (13) used a support vector machine (SVM) combined with clinical and diffusion tensor imaging (DTI) data to construct a predictive model in 160 patients with focal epilepsy. They found that the model based solely on clinical factors achieved the best performance (accuracy 78%), emphasizing that clinical variables outperformed imaging metrics in predictive importance. However, the follow-up duration was relatively short, potentially introducing bias in the conclusions. Another study (14) employed DTI-ALPS to assess glymphatic function and constructed a multiparametric prediction model in 96 pediatric epilepsy patients, achieving an AUC of 0.83. That work revealed a negative correlation between glymphatic dysfunction and seizure frequency, though the model suffered from feature redundancy. In addition, a separate study (15) calculated the graph-theoretical index of degree centrality (DC) from resting-state functional MRI (rs-fMRI) and used baseline DC maps as input features to train an SVM model, successfully achieving individualized prediction of ASM efficacy in childhood absence epilepsy (CAE) with an accuracy of 84.22%.

Overall, although the above single-modality prediction methods mostly yield AUC values in the range of 0.7–0.9 and offer the advantage of ease of implementation, traditional machine learning relies on handcrafted features, which limits the capture of complex nonlinear patterns underlying potential drug resistance (16). Therefore, the introduction of multimodal data fusion strategies is warranted to further enhance model robustness and generalizability.

### 3.2 Multimodal data fusion to enhance predictive performance

Artificial intelligence approaches based on multimodal data integrate multiple data sources—such as MRI, EEG, clinical parameters, and genetic information—thereby capturing the complex heterogeneity of epilepsy and substantially improving predictive accuracy. A growing body of evidence indicates that multimodal data fusion performs excellently in predicting drug-resistant epilepsy (DRE) or therapeutic response.

For instance, in children with epilepsy associated with tuberous sclerosis complex (TSC), Wang et al. (17) proposed a deep learning model named WAE-Net, which leverages multimodal MRI (T2WI, FLAIR) and clinical data to forecast pharmacological outcomes. This study was the first to optimize TSC lesion contrast and employed a weighted average ensemble strategy to integrate imaging and clinical information, achieving an AUC as high as 0.908. However, the investigation was constrained by a single-center, small-sample design. Similarly, Zhao et al. (18) used machine learning and statistical analysis to integrate clinical, genetic, MRI, CT, and EEG data in 103 children with TSC. Their multimodal model, which incorporated ANOVA-based feature selection and a multilayer perceptron (MLP), attained an AUC of 0.812. While the study identified MRI lesion type, EEG discharge patterns, and gene mutation type as key predictive factors, feature extraction still relied on manual annotation, introducing a degree of subjective bias. Another study in children with TSC (19) adopted an interpretable machine learning framework (random forest combined with SHAP analysis) integrating clinical, EEG, and imaging data, and achieved an AUC of 0.862 for DRE prediction in 88 patients. This model provided both global and local interpretability and incorporated a clinical nomogram to facilitate bedside application; nevertheless, the sample size was limited, and advanced imaging features were not included.

Among patients with newly diagnosed epilepsy, Yang et al. (20) combined MRI radiomic features (extracted via ResNet-18) with clinical variables using a gradient-boosting decision tree (GBDT), achieving a high predictive performance with an AUC of 0.95 in 134 patients. In temporal lobe epilepsy, Lee et al. (21) applied latent Dirichlet allocation (LDA) to extract “disease signatures” from multimodal MRI (T1, FLAIR, DWI) in 82 patients to predict medication response (balanced accuracy 76%). This approach effectively captured continuous variation in disease manifestation, outperforming conventional analytical methods; however, the sample size was modest and the analysis relied solely on structural imaging without incorporating functional data.

Collectively, these studies suggest that multimodal fusion can improve feature representation and may increase task-specific predictive performance. However, the reported AUC values arise from different populations, endpoints, sample sizes, and validation designs and should not be compared directly. Most studies remain limited by small, single-center cohorts; external validation and interpretable analyses, such as SHAP, are therefore priorities for clinical translation. The multiscale biological determinants of ASM response and their corresponding multimodal signatures are summarized in Figure 2.

**Figure 2:**
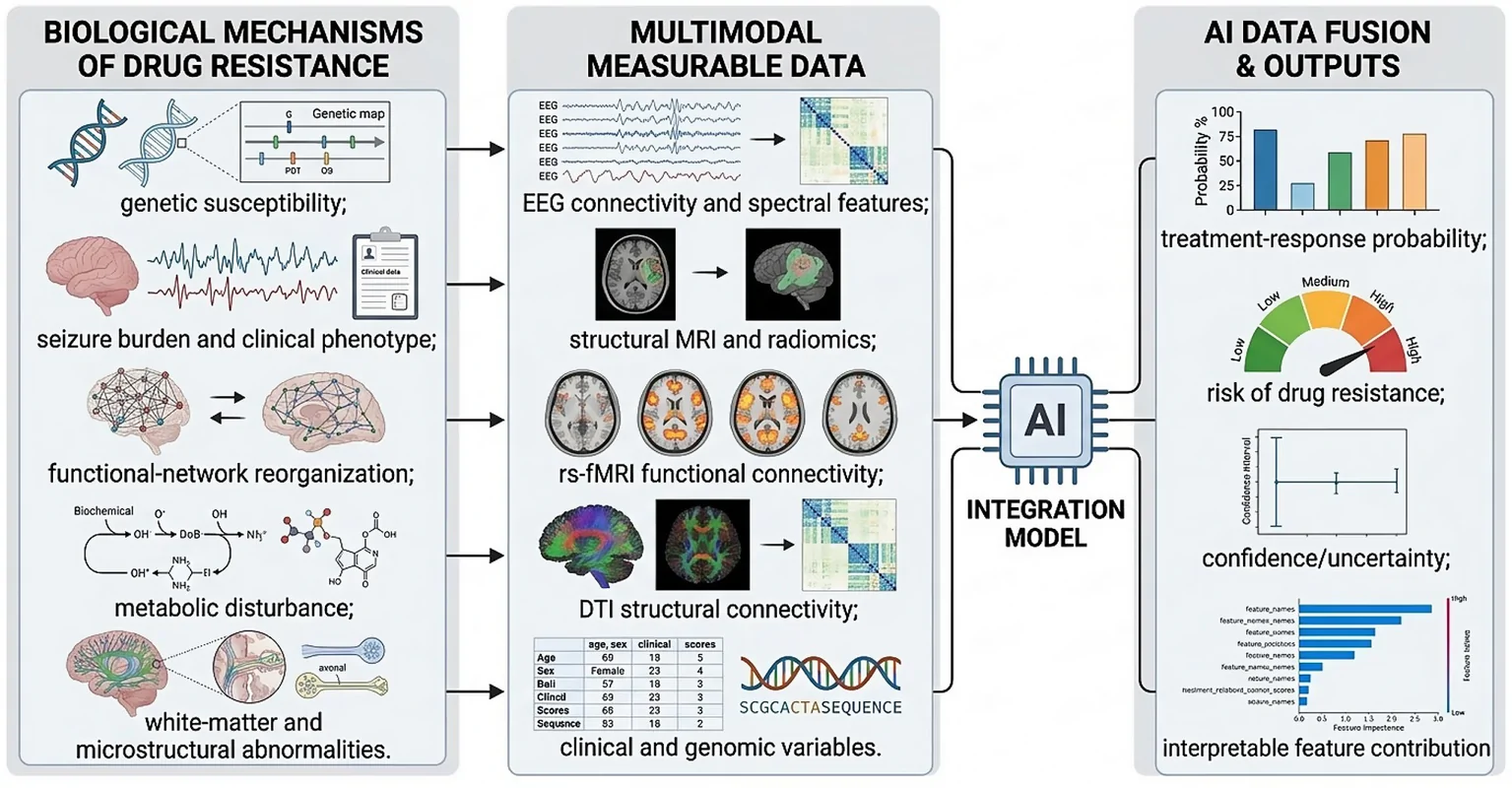
Multimodal framework for predicting anti-seizure medication response. Genetic susceptibility, seizure burden, network reorganization, metabolic disturbance, and microstructural abnormalities generate complementary EEG, imaging, clinical, and genomic features. Multimodal AI integrates these signals to estimate treatment response, drug-resistance risk, predictive uncertainty, and interpretable feature contributions.

### 3.3 Complementary value of clinical and genetic data

Although advanced imaging and EEG features play a prominent role in predicting response to antiseizure medications (ASMs), non-imaging approaches based on clinical, genetic, or electronic medical record data retain substantial practical value, particularly in resource-limited settings or for population-based screening, owing to their ready accessibility and feasibility for large-scale deployment.

For example, a 2021 study (22) integrated clinical information with whole-genome sequencing (WGS) data and employed the XGBoost algorithm to predict patient response to brivaracetam, achieving an AUC of 0.76 in a cohort of 235 patients. This study identified potential genetic biomarkers, including structural variants near the SV2A gene and mutations in microtubule-related gene sets. In 2022, a multicenter study (23) (*n* = 1,798) used a Transformer model to predict response to the initial ASM based solely on 16 clinical features (including seizure frequency >5 episodes, EEG abnormalities, and history of psychiatric disorders), attaining an AUC of 0.65. Although the model supported individualized predictions and was made open-source, its generalizability remained insufficient. In a study of patients with familial genetic generalized epilepsy (GGE), Wu et al. (24) utilized a random forest model in 854 patients and achieved a prediction accuracy of 91% for treatment response, identifying “number of seizure types” as the most important predictor; however, this study was limited by class imbalance issues.

Furthermore, predictive models based on large-scale real-world data—such as medical insurance claims and electronic health records—have also shown promising application potential. A 2018 study (25) leveraged claims data from 290,000 patients in the United States to construct a random forest model that predicted DRE risk at the time of the first prescription, achieving an AUC of 0.764 and providing risk warnings on average two years in advance. Another study (26) based on electronic medical records (EMR) employed a gradient-boosted tree (GBT) algorithm for DRE identification, achieving an AUC of 0.829—a performance significantly superior to the initial diagnostic accuracy of physicians (9.5%).

## 4 AI-based auxiliary localization of epileptogenic foci

For patients with drug-resistant epilepsy (DRE), surgical resection of the epileptogenic zone (EZ) remains one of the most effective treatment modalities; its success critically depends on the precise pre-surgical identification and delineation of the epileptogenic region. However, the EZ itself cannot be directly and accurately demarcated by existing techniques (27); current imaging and EEG methods can only identify functional and structural abnormalities that are spatiotemporally coordinated with the EZ, and infer the seizure focus based on such indirect evidence. Against this background, the development of more precise EZ localization techniques—particularly automated multimodal analysis based on artificial intelligence—has become a key focus of current research.

### 4.1 Single-modality artificial intelligence approaches

Single-modality AI methods for epileptogenic focus localization mainly focus on a specific data type (e.g., PET, EEG, or fMRI). Although multimodal fusion can provide more comprehensive information, single-modality approaches retain significant research value and practical application potential due to their relatively easier data acquisition, more straightforward processing workflows, and lower barriers to clinical integration. In recent years, driven by marked advances in deep learning, feature engineering, and signal processing, numerous intelligent localization methods based on a single imaging modality—such as scalp or intracranial EEG, structural or functional MRI, and FDG-PET—have emerged. Models including CNNs, SVMs, and Naïve Bayes are widely used for feature extraction and classification tasks, enabling automated processing from signal source imaging to three-dimensional visualization. These methods not only compensate for the limitations of traditional visual analysis, but also demonstrate important adjunctive diagnostic value in complex clinical scenarios such as MRI-negative epilepsy, pediatric epilepsy, and extra-temporal lobe epilepsy.

#### 4.1.1 EEG-based localization methods

EEG (including scalp and intracranial recordings) is the core electrophysiological tool for epilepsy diagnosis and EZ localization, and its high temporal resolution offers a unique advantage for dynamically capturing epileptic discharges. To date, extensive research has been conducted on automated EZ localization using EEG signals, with these methods focusing primarily on the automatic identification of the seizure onset zone (SOZ) through the analysis of interictal signals. In recent years, AI-based EEG approaches have concentrated on the following directions:

1. Interictal EEG source imaging and functional connectivity analysis. A 2024 study published in Epilepsia (28) used 19- to 25-channel scalp EEG, reconstructed cortical electrical activity via electrophysiological source imaging (ESI), and combined functional connectivity (FC) features with a machine learning model to achieve an accuracy of 75% in individualized EZ localization, with the high-gamma band performing best. Similarly, another study employed sLORETA source imaging, introduced time-frequency analyses (29)—short-time Fourier transform (STFT), continuous wavelet transform (CWT), and superlets—in combination with a VGG-16 network, and achieved a localization accuracy of 83.1% on 16-channel EEG data, indicating that time-frequency features and deep learning retain considerable potential even with a limited number of channels.
2. Integration of intracranial EEG (iEEG/SEEG) biomarkers and dynamic analysis. Multiple studies have focused on extracting diverse biomarkers from iEEG (e.g., sharp waves, high-frequency oscillations [HFOs], phase-amplitude coupling [PAC], network connectivity features) and using machine learning for automated SOZ localization. For instance, a 2024 Epilepsia study (30) reported that using only low-frequency SEEG features below 45 Hz achieved a localization performance comparable to that of the full frequency band, challenging the conventional notion that high-frequency oscillations are irreplaceable. A 2025 study (31) adopted the perspective of “pre- and post-ictal dynamic modulation,” integrating PAC, directed transfer function (DTF), and nonlinear permutation entropy features, and attained an AUC of 0.905 in SOZ localization using an XGBoost model.
3. Introduction of cybernetics and causal network models. A 2025 study (32) applied, for the first time, the cybernetic metric of “dynamic functional network controllability” to iEEG analysis, achieving an *F*1-score of 80.25% in the low-gamma band, outperforming traditional graph-theoretical and power features. Another study (33) proposed a causal influence index (CII) based on phase transfer entropy (PTE) to construct causal networks, and combined with a random forest model reached an SOZ identification accuracy of 82.18% and an AUC of 0.90, highlighting the important roles of network control and causal inference in epilepsy localization.

#### 4.1.2 MRI-based localization methods

In addition to EEG, structural MRI plays an irreplaceable role in detecting structural lesions such as FCD and TSC nodules. In recent years, deep learning models based on a single MRI sequence have made notable progress in lesion detection and segmentation. For example, a 2024 study (34) employed a 3D full-resolution nnU-Net on T1-MPRAGE images from 65 children with FCD type II to achieve fully automated lesion segmentation, with a Dice coefficient of 0.57. This model did not rely on FLAIR sequences or complex cortical reconstruction pipelines, demonstrating the potential of deep learning in single-sequence image analysis. Another study (35) targeted FLAIR-negative FCD patients and proposed a CNN framework based on activation maximization and convolutional matching (AMCL), achieving a recall of 83.33% in 19 patients, indicating that even in radiologically negative cases, AI models can still effectively identify subtle morphological abnormalities.

#### 4.1.3 PET- and fMRI-based localization methods

PET and fMRI focus on metabolic or functional abnormalities. Wang et al. (36) used 19 quantitative PET metrics (including SUVmean, TLG, and normalized metabolic activity) to construct a logistic regression model for predicting epileptogenic tubers in TSC patients, achieving an AUC of 0.85 on an external test set, and demonstrated good clinical utility through nomogram visualization and decision curve analysis. Another study (37) performed cortical surface analysis on FDG-PET images and systematically compared for the first time the performance of cortical thickness, sulcal depth, and gyral index in FCD diagnosis; they found that SVM-based cortical thickness classification achieved an accuracy of 95.5%, but the consistency in accurately localizing the EZ remained limited (Kappa = 0.6). In addition, a 2022 study (38) innovatively employed a “zero-shot” learning paradigm, using resting-state fMRI data from 2,132 healthy controls to simulate noise for training a 3D CNN model, and achieved a lateralization accuracy of 90.6% in 32 patients with unilateral temporal lobe epilepsy (TLE). This approach partially alleviated the issue of scarce patient data, but its focus remained only on lateralization without achieving precise EZ localization.

#### 4.1.4 Overall performance of single-modality approaches

Overall, most studies have reported good localization performance in independent testing or cross-validation: EEG methods generally achieve accuracies ranging from 75 to 90%, MRI segmentation Dice coefficients mostly fall between 0.5 and 0.6, and PET classification models can reach AUCs of 0.85–0.93. Although single-modality AI approaches have made substantial progress in epileptogenic focus localization, they still commonly suffer from the limitations of single-center designs and small sample sizes. From a technical perspective, EEG methods face challenges such as limited channel numbers, low spatial resolution, and high computational complexity; MRI methods are constrained by high lesion heterogeneity and difficulty in detecting negative samples; and PET or rs-fMRI approaches depend heavily on image quality and exhibit diverse abnormal patterns, making stability a challenge. Furthermore, in clinical integration, high false-positive rates and insufficient model interpretability also hinder their widespread adoption.

Despite these challenges, single-modality AI-based epileptogenic focus localization methods have achieved notable advances across the three major modalities of EEG, MRI, and PET. Through innovative feature extraction, dynamic analysis, symmetry learning, and cybernetic modeling, these techniques have achieved multidimensional progress—from signal to imaging, from pediatric to adult populations, and from detection to prediction. As algorithms continue to be optimized and clinical collaboration deepens, single-modality AI tools are expected to become efficient and reliable auxiliary means in the pre-surgical evaluation of epilepsy, holding particular research value and clinical application prospects in resource-limited settings or when data are incomplete.

### 4.2 Multimodal artificial intelligence approaches

Conventional focus localization relies on single-modality techniques such as EEG/iEEG, MRI, PET, and SPECT. An automated focus localization pipeline typically encompasses preprocessing, feature extraction, classification, and evaluation, which can reduce dependence on manual interpretation and improve diagnostic efficiency. However, a single modality often suffers from insufficient sensitivity or specificity. For example, functional abnormalities and structural abnormalities do not completely overlap, adding to the uncertainty of localization. With the rapid advancement of artificial intelligence, particularly deep learning, automated analytical models that integrate multimodal imaging data have gradually become a research hotspot. Such approaches can synthesize the complementary information provided by different imaging modalities—including the anatomical details from structural MRI, white-matter connectivity from DTI, metabolic information from PET, and electrophysiological dynamics from sEEG/iEEG—and extract epileptogenic biomarkers in a data-driven manner, thereby achieving more objective and accurate focus localization.

From a methodological perspective, AI models based on multimodal brain imaging can be broadly classified into the following categories.

#### 4.2.1 Methods based on intracranial EEG and multimodal fusion

This category primarily employs intracranial EEG and structural imaging as the main modalities, integrating electrophysiological, structural, and spatial features via graph neural networks or conventional machine learning to achieve high-resolution EZ identification and surgical planning. For example, a 2025 study (39) developed a framework based on a graph attention network (GAT) that treated sEEG electrode contacts as nodes and fused electrophysiological features, spatial coordinates, and gray/white matter probability features, significantly improving EZ identification accuracy (AUPRC = 0.69). This study was the first to explicitly encode the “electrode implantation topology” as a graph structure; however, it encountered difficulties in handling bimodal or multifocal epilepsy cases. Similarly, another study (40) proposed the ST-CANe multimodal fusion model, which integrated sEEG spatiotemporal features with DTI structural connectivity features via a co-attention mechanism, achieving a Dice similarity coefficient of 95.65% in temporal lobe epilepsy. This model was the first to systematically fuse sEEG electrophysiological signals with DTI structural connectivity, but the sample size was small (only 15 patients), and generalizability requires further validation. In addition, Horsley et al. (41) combined dMRI connectivity abnormalities and iEEG functional abnormalities to localize the EZ in patients with drug-resistant focal epilepsy, and found that concurrent resection of the regions with the most prominent abnormalities in both categories significantly improved postoperative seizure-freedom rates. Although that study used normative atlas comparisons to enhance objectivity, it still had limitations such as a small sample size and retrospective design.

#### 4.2.2 Lesion detection and segmentation based on multimodal structural MRI

These methods utilize structural MRI data from multiple sequences (e.g., T1-weighted, T2-weighted, FLAIR) or incorporate diffusion imaging techniques to achieve precise localization in refractory epilepsy, and are widely applied to the identification of structural lesions such as focal cortical dysplasia (FCD) (42–44) and tuberous sclerosis complex (TSC) (45, 46). Sun et al. (45) conducted a prospective study to evaluate the combined value of DTI (fractional anisotropy [FA], axial diffusivity [AD], etc.) and MAP-MRI (mean squared displacement [MSD], quartic inversion coefficient [QIV], etc.) in non-invasively identifying epileptogenic cortical tubers in children with TSC. Furthermore, deep learning architectures such as CNN (43, 47), U-Net (46), FCN (42), and msResNet (48) have been employed to process multi-sequence MRI data (T1, T2, FLAIR). A 2025 multicenter comparative study (49) demonstrated that 3D-nnUNet achieved the best overall performance in FCD detection (*F*1-score = 0.58), whereas deepFCD, despite high sensitivity (82%), exhibited relatively low specificity. These findings indicate that multi-sequence MRI fusion strategies can significantly improve the detection rate in MRI-negative cases.

#### 4.2.3 Metabolic-structural fusion analysis based on PET and MRI

^18^F-FDG PET can demarcate the EZ using ictal hypermetabolism and interictal hypometabolism, but its limited spatial resolution necessitates fusion with structural imaging to precisely delineate lesion boundaries. A 2018 study (50) was the first to systematically integrate MRI-based surface morphological features with PET metabolic features, extracting multimodal metrics at cortical surface vertices and combining SVM with a non-local means fusion strategy, which increased the FCD detection rate to 93%. However, the control group in that study comprised patients with MRI-negative temporal lobe epilepsy (TLE) rather than healthy individuals, which may have been influenced by seizure-related metabolic or structural alterations. Another study (51) further proposed a “cortical basis mapping” (CBM) pipeline, which was the first to systematically apply a “surface-based unified SUVR Z-transform” method to MRI-negative epilepsy, circumventing conventional voxel-level registration errors and achieving a localization concordance rate of 87%, significantly outperforming the traditional SPM approach. In addition, Mareček et al. (52) used a Gaussian mixture model (GMM) for unsupervised feature learning and classification, exploring multimodal fusion of MRI, PET, and SPECT. Lin et al. (53) developed a surface-based cortical feature classifier that integrated T1-weighted images, FLAIR MRI, and PET images to accurately localize FCD type IIb lesions; their results showed that the classifier trained with combined PET and MRI features achieved the best performance. The partially overlapping biological signatures captured by different modalities and their convergence in AI-assisted EZ localization are illustrated in Figure 3.

**Figure 3:**
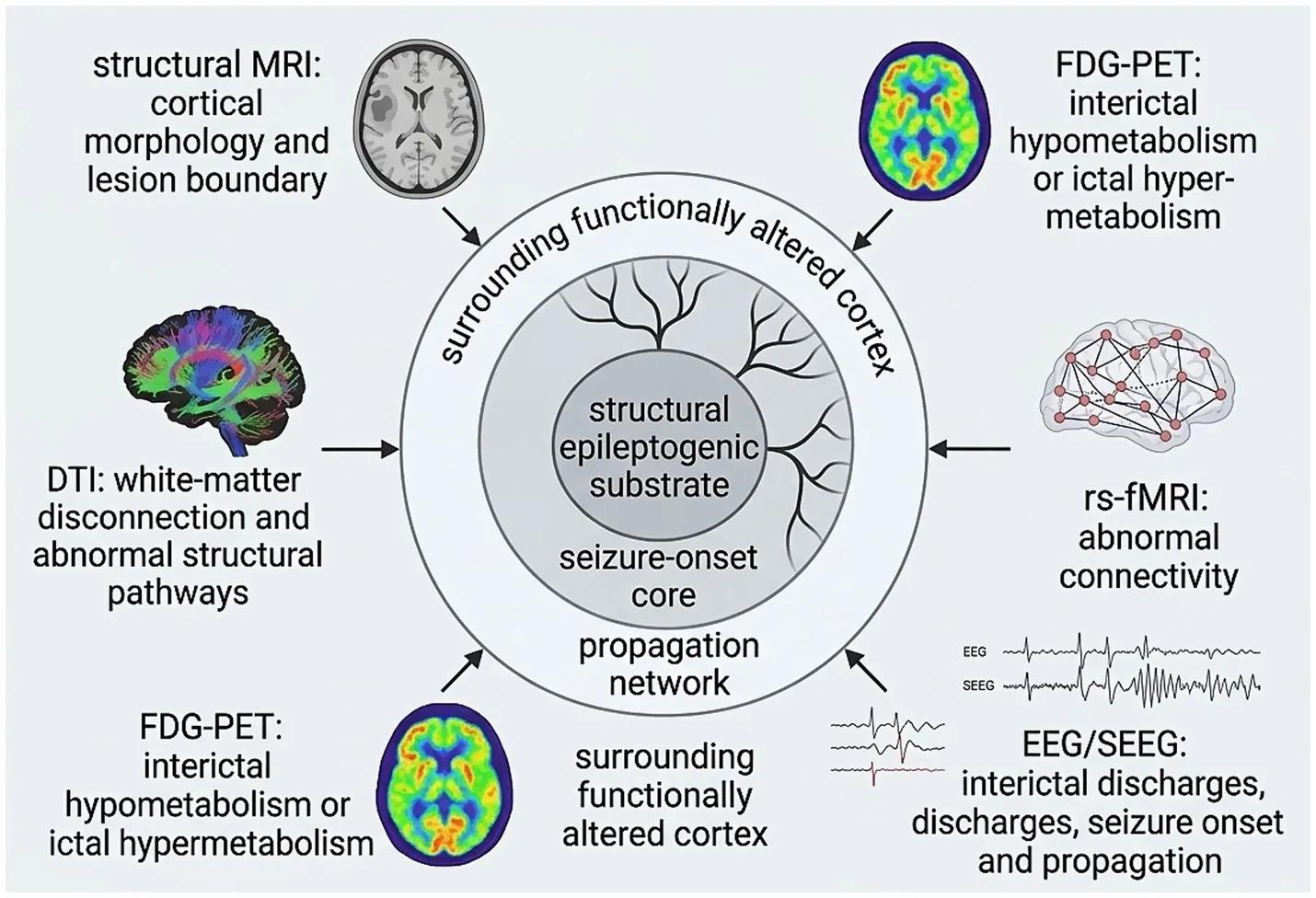
Multimodal characterization of the epileptogenic network. Structural MRI, DTI, FDG-PET, rs-fMRI, and EEG/SEEG provide complementary information on the structural epileptogenic substrate, seizure-onset core, propagation network, and surrounding functionally altered cortex, supporting integrated epileptogenic-zone localization.

#### 4.2.4 Virtual brain models and computational modeling

This category constructs personalized virtual brain models (54) to estimate the EZ through computational modeling and perform virtual surgery simulations to assist clinical decision-making. A research team from Aix-Marseille University proposed a novel workflow called the Virtual Epileptic Patient (VEP) (55–57), which integrates T1-MRI, dMRI, and sEEG data and constructs a dynamic network model via Bayesian inference. This model can not only identify the EZ but also predict the outcomes of virtual resections. The VEP model has entered the validation phase in multiple prospective clinical trials, demonstrating considerable theoretical innovation and clinical application potential; however, it still faces challenges such as high computational resource requirements and dependence on high-quality data. The mechanistic sequence from patient-specific connectome construction to seizure simulation and virtual resection is shown in Figure 4.

**Figure 4:**
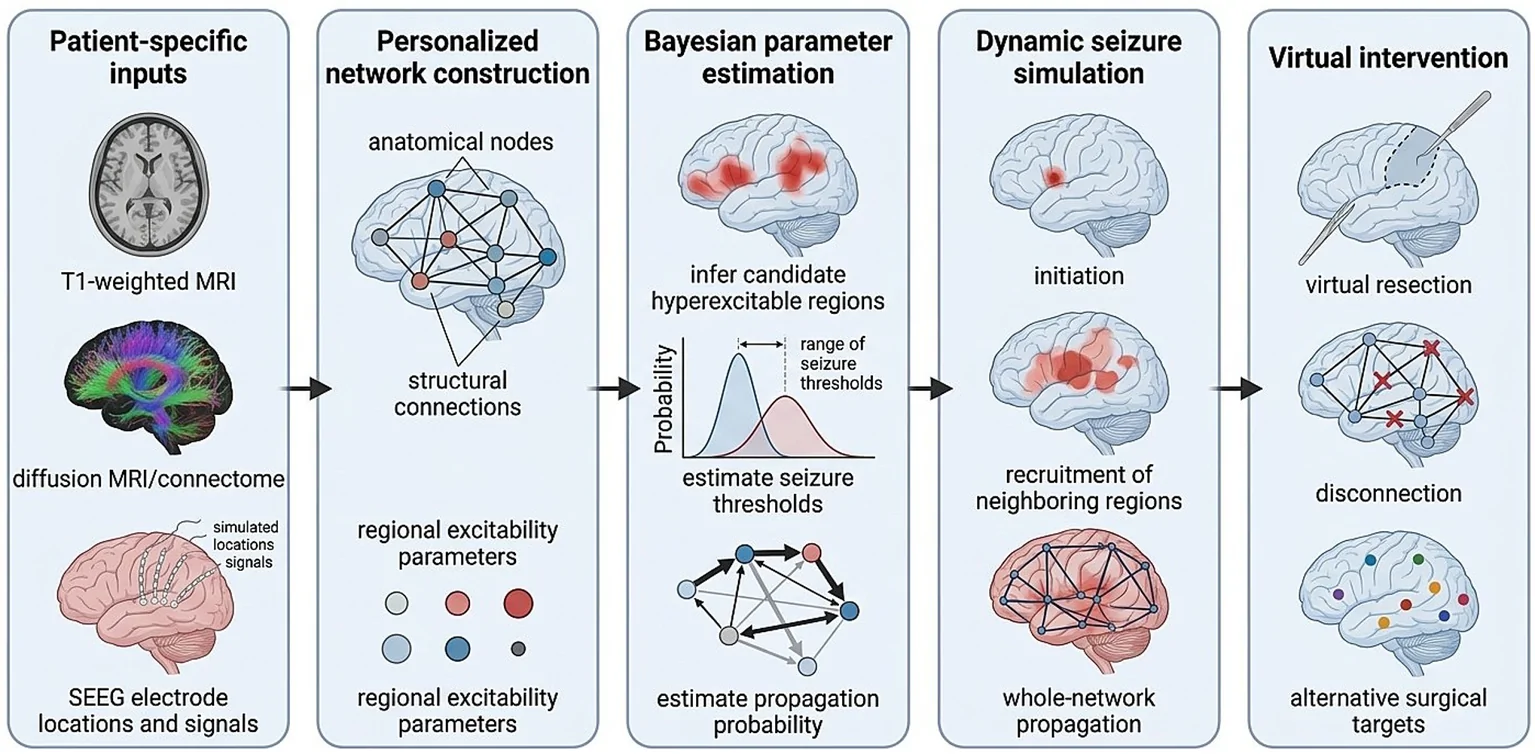
Personalized virtual brain modeling for epilepsy surgery. Patient-specific structural MRI, diffusion connectomics, and SEEG data are integrated to construct individualized brain networks. Bayesian parameter estimation and dynamic seizure simulation are then used to identify hyperexcitable regions, model seizure propagation, and evaluate virtual resection or disconnection strategies.

#### 4.2.5 Overall performance of multimodal fusion approaches

AI methods based on multimodal brain imaging have shown promising sensitivity and spatial concordance for selected EZ-localization tasks. In some MRI-negative cohorts, multimodal fusion increased lesion-detection rates, supporting an adjunctive role in presurgical planning. These findings remain task- and cohort-specific and require prospective external validation before routine clinical use.

Nevertheless, multimodal brain imaging AI approaches still face several challenges in epilepsy localization applications. First, data quality and annotation consistency are critical factors affecting model generalizability. Most current studies are based on single-center, small-sample data (*n* < 100), and differences across institutions in imaging protocols, scanning parameters, and physician annotation standards lead to unstable model performance. Second, the high computational complexity of some models limits their clinical translation. Third, false-positive issues remain prominent, particularly in anatomically complex regions such as the insula, hippocampus, and temporal pole, where mislocalization may interfere with clinical decision-making. In addition, existing models are mostly optimized for specific pathological types, and their generalizability to other epilepsy subtypes remains to be validated.

Future research should focus on constructing large-scale, multicenter epilepsy imaging datasets with standardized annotation protocols, developing lighter-weight, more efficient, and interpretable AI architectures, further deepening multimodal information integration, and strengthening prospective clinical validation. Such efforts will drive a fundamental shift in epilepsy localization from an experience-dependent paradigm toward a precision medicine model.

### 4.3 Methods based on generative models, data augmentation, and innovative modalities

Although multimodal and unimodal artificial intelligence approaches have achieved considerable progress in epileptogenic focus localization, they still face multiple challenges in clinical practice, including scarcity of training data, noisy annotations, incomplete modalities, and high disease heterogeneity. In recent years, with the continuous advancement of artificial intelligence in this field, a growing number of studies incorporating generative models, data augmentation, novel signal modalities, and natural language processing have emerged, offering new solutions for focus localization. These approaches not only broaden the technical pathways but also effectively enhance model adaptability and robustness in complex clinical environments.

#### 4.3.1 Application of generative models and data augmentation in epilepsy localization

Generative models and data augmentation techniques mitigate the issues of scarce clinical data and variable image quality by synthesizing data or improving image quality. Generative adversarial networks (GANs) have been employed to generate “normal” PET images and perform difference analyses, reducing reliance on large-scale normative templates. For example, a 2023 study proposed the SIPCOM method (58), which used a conditional GAN to generate theoretically “normal” FDG-PET images from patients’ T1-weighted MRI, and then obtained difference *Z*-score maps by subtracting real PET images. In 20 patients, this method achieved a resection zone localization rate of 60%, significantly outperforming the conventional SPM approach, and provided a new metabolic comparison strategy for MRI-negative epilepsy that does not depend on healthy-subject templates. Another study (59) utilized a Monte Carlo simulator to generate large volumes of realistic PET data, and combined this with a 3D ResNet for image super-resolution reconstruction, increasing the visual detection rate from 38 to 75% in 10 epilepsy patients. This method markedly improved PET image contrast and lesion detectability without relying on MRI, making it particularly suitable for resource-limited clinical settings.

Notably, researchers from the University of Wisconsin have proposed multiple augmentation-based localization frameworks using resting-state fMRI data from healthy individuals. In a 2023 study (60), they implanted noise-simulated epileptogenic zones into healthy-subject rs-fMRI data to train deep learning models for zero-shot or few-shot epilepsy localization. The proposed dynamic functional connectivity (dFC) combined with a GCN-Transformer framework achieved an AUC of 0.77 in 14 pediatric patients, significantly outperforming static methods. In 2024, the same team (61) further introduced a label-noise modeling network that jointly trained noise representation and localization tasks, improving the AUC from 0.57 to 0.71 on simulated data, demonstrating its potential in handling clinical annotation uncertainty.

#### 4.3.2 Innovative signal modalities and quantitative imaging techniques

In addition to conventional EEG and MRI, several novel signal modalities and imaging techniques that have not yet been widely adopted—but hold considerable potential—are providing new biomarkers and solutions for epileptogenic focus localization.

Magnetic resonance fingerprinting (MRF) is a novel quantitative MRI technique that can simultaneously quantify multiple tissue properties within a relatively short scan time. A 2022 study (62) was the first to apply MRF to subcortical nucleus analysis in epilepsy patients, finding significantly elevated ipsilateral thalamic T1 values, and constructed a support vector machine (SVM)-based classification model that achieved an AUC of 0.83 in 48 patients. A 2025 study (63) further combined MRF with nnU-Net to achieve automated whole-brain detection of focal cortical dysplasia, with a sensitivity of 80%; even in MRI-negative cases, the detection rate remained at 67%, significantly outperforming the conventional MAP18 method.

Cortico-cortical evoked potential (CCEP) mapping is a rapidly evolving technique for brain network visualization and cortical excitability assessment, aiding in the identification of causal relationships between brain regions in information processing. A 2022 study (64) was the first to apply machine learning to 650,000 CCEP recordings, achieving an AUC of 0.83 for SOZ localization using a soft-voting ensemble model in seven patients. Another study (65) used single-pulse electrical stimulation (SPES)-evoked potentials from SEEG to train a one-dimensional convolutional neural network, achieving a sensitivity of 78.1% in ten patients with temporal lobe epilepsy, indicating that short stimulation sessions may complement long-term monitoring in selected settings.

In addition, network control theory and dynamic effective connectivity analysis have provided new perspectives for epilepsy localization. A 2024 study published in Epilepsy Research (66) combined average controllability (AC) from network control theory with graph-theoretical features, and employed a dung beetle optimizer to optimize an SVM model, achieving a high AUC of 0.96 in 12 patients, highlighting the notable advantages of controllability features in identifying epileptogenic nodes. Another study (67) constructed high-frequency oscillation (HFO) and seizure propagation networks, and via unsupervised graph convolutional network (GCN) clustering achieved an SOZ recall of 90.35%. Although the precision was relatively low (44.83%), it still substantially narrowed the scope for manual clinical detection.

#### 4.3.3 Natural language processing and semiological analysis

The introduction of natural language processing (NLP) techniques has opened new research directions for text-based epilepsy localization. A 2024 study published in Scientific Reports (68) focused on semi-structured Italian epilepsy text descriptions, using a feature representation combining bag-of-words (BoW) and Word2Vec embeddings, and constructed an SVM-based classification model. The model achieved an F1 score of 85.6% in distinguishing temporal from extra-temporal lobe epilepsy, but lateralization performance was relatively poor (68.5%), suggesting that extracting lateralizing information embedded in textual descriptions remains challenging. A 2025 study (69) further evaluated the capability of ChatGPT-4 in interpreting ictal semiology, and found that its weighted sensitivity (67–74%) in both public and private cohorts was significantly higher than that of human experts (49–51%), with particularly strong performance in localizing frontal and temporal lobe regions. Although performance was limited in rare brain regions (e.g., cingulate gyrus, insula) and some geographical bias was observed, its potential as a clinical adjunctive tool has been preliminarily validated.

Overall, emerging techniques including generative models, data augmentation, innovative modalities, and natural language processing have injected new momentum into epileptogenic focus localization research. These methods have achieved multidimensional technical breakthroughs, ranging from image synthesis and signal parsing to text comprehension, and have demonstrated potential in clinical applications as potential complements to conventional approaches. Although challenges remain in data quality, algorithmic reliability, and clinical integration, continued optimization and deepening interdisciplinary collaboration are expected to position these innovative tools as integral components of the next-generation pre-surgical epilepsy evaluation system, offering more precise, efficient, and personalized diagnostic support for a broader population of patients with drug-resistant epilepsy.

## 5 AI-based prediction of surgical outcomes in epilepsy

Epilepsy surgery represents an important treatment option for patients with drug-resistant epilepsy (DRE); its success directly affects patients’ quality of life and long-term neurological function. However, surgical outcomes exhibit substantial interindividual variability, and even among rigorously selected patients, a considerable proportion fail to achieve postoperative seizure freedom or experience varying degrees of neurological deficits. Therefore, accurate preoperative prediction of surgical outcomes has become one of the core challenges in the field of epilepsy surgery. Traditional prediction largely relies on clinical experience and single-modality indicators, which are highly subjective and limited in accuracy. In recent years, with the rapid advancement of artificial intelligence—particularly the strengths of machine learning and deep learning in multimodal data integration and pattern recognition—novel avenues have emerged for constructing individualized, high-precision models for surgical outcome prediction. This section reviews the application of artificial intelligence in predicting epilepsy surgery outcomes, analyzes various approaches including multimodal, unimodal, neuropsychological, intraoperative assistive, and natural language processing methods, discusses their technical progress and clinical value, and explores current challenges and future directions.

### 5.1 Unimodal artificial intelligence approaches

Among prediction studies based on unimodal data, structural brain network abnormalities revealed by diffusion MRI (dMRI) have become a research hotspot. Predictive models constructed using graph-theoretical metrics (e.g., betweenness centrality [BC]) (70) achieved an AUC of 0.88 in multicenter temporal lobe epilepsy (TLE) data and demonstrated stable performance in independent test sets, indicating that hub regions in structural networks hold significant predictive value for surgical prognosis. Deep learning models have also been applied to high-dimensional connectome data; for example, one study used dMRI to construct 384 × 384 connectivity matrices and employed deep learning-generated binary masks for dimensionality reduction to predict postoperative outcomes in TLE, achieving a positive predictive value of 88%. Another study focused on pediatric language function prognosis (71), using a deep convolutional neural network (DCNN) to classify fiber tracts and construct a language module network, achieving a prediction accuracy of 96%. These findings highlight the importance of brain network analysis in prognostic prediction; however, the computational complexity remains high, and most approaches still rely on unimodal data.

Intracranial EEG and scalp EEG have also been widely used for surgical outcome prediction. Some studies have extracted high-frequency coherence features from visually normal scalp EEG (72), demonstrating superior performance over MRI-based indicators. Others have utilized peri-ictal scalp EEG features (73) with a LightGBM model to achieve an accuracy of 91.9% and an AUC of 0.977, with decision curve analysis showing significant clinical net benefit. In terms of intracranial EEG, research has focused more on deep-level features such as causal networks and high-frequency oscillations. For example, causal connectivity based on the FCCM algorithm (74) showed significant differences in the alpha band, and when combined with SVM achieved a prediction accuracy exceeding 87%; a genetic neural network (GNN) combined with hybrid iEEG markers (75) further reached an accuracy of 94.3% in 79 patients.

In addition, structural MRI has been used to predict surgical outcomes. Automated volumetric analysis tools (e.g., NeuroQuant) (76, 77) can rapidly assess volume loss in frontal or temporal cortices; when combined with clinical indicators, the predictive model achieved an AUC of 0.897, demonstrating good potential for clinical translation.

### 5.2 Multimodal artificial intelligence approaches

In multimodal data-driven AI prediction studies, the integration of structural MRI, FDG-PET, and postoperative resection information has been shown to significantly improve model performance. A 2022 study (78) extracted features including temporal hypometabolic volume, hippocampal sclerosis markers, and resection extent, and employed algorithms such as random forest and support vector machine to predict postoperative outcomes in mesial temporal lobe epilepsy (MTLE) patients, achieving a model AUC of 0.81. This study particularly emphasized the critical role of postoperative resection information: when relying solely on preoperative features, model performance declined substantially (AUC dropping to 0.59–0.62). In pediatric epilepsy surgery prediction, a multicenter study (79) used algorithms including XGBoost to integrate clinical, surgical, and diagnostic features (e.g., MRI lesion type, video-EEG concordance), developing a practical prediction table that outputs individualized seizure-freedom probabilities; however, PET and magnetoencephalography (MEG) contributed limitedly due to high rates of missing data. Notably, methods such as Bayesian networks have demonstrated advantages in handling missing values in real-world clinical data—for instance, the MBC model (80) achieved AUCs exceeding 0.67 for 1-year, 2-year, and 5-year outcomes in TLE, and has been developed into an online tool for clinical use.

Models with superior performance have often relied on deep learning. For example, one study (81) used a pre-trained ResNet-34 to extract features from multimodal neuroimaging (MRI, PET, CT), and then combined a multi-kernel SVM with an attention mechanism, achieving an accuracy of 91.49% and an AUC of 0.952 in 141 patients. However, this model did not incorporate electrophysiological data, and its generalizability requires further validation. Beyond conventional features, brain network metrics have also shown considerable potential: studies have identified a “functional connectivity contraction” phenomenon (82) in TLE patients; machine learning models based on this feature achieved an accuracy of 76% in predicting seizure-free outcomes, significantly outperforming models relying solely on clinical and structural features (58%). In addition, unsupervised learning methods such as latent Dirichlet allocation (LDA) (21) have extracted disease factors from multimodal MRI, achieving a balanced accuracy of 88% in surgical outcome prediction.

### 5.3 Other atypical indicators

Beyond traditional seizure outcome prediction, AI techniques have also been applied to atypical prognostic indicators. For instance, natural language processing (NLP) has been used to automatically extract clinical information from electronic medical records (83, 84), either to quantify semiology-imaging concordance for assisting EZ localization (although not significantly improving outcome prediction) or to automatically identify patients eligible for surgical referral, with the model achieving an AUROC of 0.97 for this task. Furthermore, a nomogram tool has been developed to predict postoperative mood decline (85) (*C*-statistic = 0.70), and a random forest model has been constructed to predict short-term non-epilepsy outcomes (86) (e.g., prolonged hospitalization, non-home discharge) with AUROCs ranging from 0.74 to 0.86; this model has been deployed as a web application to support rapid clinical assessment.

In functional outcome prediction, task-related high-gamma activity from iEEG recordings *γ* (87) has been shown to predict changes in postoperative language scores (*r*^2^ = 0.32–0.50), while machine learning models have achieved an accuracy of 80% in predicting postoperative language decline.

Overall, artificial intelligence has substantially improved the accuracy (70–90%) of surgical outcome prediction in epilepsy. Multimodal approaches effectively capture complex brain network abnormalities, unimodal methods lower the barrier to clinical application, and generative models, natural language processing, and biotyping techniques further enhance individualized prediction capabilities. Open-source tools (e.g., MBC, nnU-Net) have also facilitated the dissemination and translation of relevant algorithms. Table 2 summarizes representative studies across the three clinical tasks. Because the cohorts, endpoints, validation strategies, and reported metrics differ, these values should not be used for direct cross-study or cross-task ranking.

**Table 2:** Representative evidence for multimodal and AI-assisted epilepsy prediction tasks.

Study	Task	*n*	Input data	Model	Validation	Reported metric
Yang et al. (11)	ASM response	101	Clinical + EEG	1D-CNN	Split sample + 10-fold CV	AUC 0.81; validation accuracy 0.84
Wang et al. (17)	ASM response in TSC	300	T2WI + FLAIR + clinical	WAE-Net	Independent test cohort	AUC 0.908
Zhao et al. (18)	ASM response in TSC	103	Clinical + genetic + MRI + CT + EEG	MLP	Internal validation	AUC 0.812
Yang et al. (20)	Early DRE identification	134	MRI radiomics + clinical	ResNet-18 + GBDT	Internal validation	AUC 0.95
Zhang et al. (34)	FCD localization	65	T1-MPRAGE	3D nnU-Net	Internal test set	Dice 0.57
Luckett et al. (38)	TLE lateralization	32	rs-fMRI	3D CNN	Patient-level testing	Accuracy 0.906
Xiao et al. (40)	EZ localization	15	sEEG + DTI	ST-CANe	Internal validation	Dice 0.9565
Tang et al. (81)	Surgical outcome	141	MRI + PET + CT	ResNet-34 + multi-kernel SVM	Internal validation	AUC 0.952; accuracy 0.9149

## 6 Discussion

In recent years, artificial intelligence techniques based on brain imaging have made significant progress in the field of epilepsy-assisted diagnosis and treatment, demonstrating notable advantages in key areas such as epileptogenic focus localization, prediction of medication response, and surgical outcome assessment. These advances reveal a promising trajectory from traditional experience-based interpretation toward data-driven precision medicine. Current research has evolved from early single-modality analyses to multimodal fusion and intelligent decision support, encompassing structural, functional, metabolic, and electrophysiological dimensions, and has preliminarily established a research framework centered on data and oriented toward clinical translation. By integrating multiple neuroimaging and electrophysiological modalities including structural/functional MRI, PET, EEG/iEEG, DTI, and MEG and combining them with machine learning (ML) and deep learning (DL) algorithms, multimodal AI can uncover cross-modal, multidimensional associative features, significantly enhancing diagnostic efficiency, localization accuracy, and prognostic capability in epilepsy care.

### 6.1 Limitations of current research

Although AI techniques have demonstrated substantial potential in epilepsy—particularly in the detection and prediction of focal cortical dysplasia (FCD)—the pathway from algorithm development to clinical application remains fraught with challenges. The core difficulties confronting multimodal brain-imaging AI research span the entire process of data acquisition, model development, evaluation, and clinical integration.

#### 6.1.1 Data acquisition and quality

Epilepsy, as a complex and severe brain disorder, inherently suffers from limited sample sizes, especially for certain FCD subtypes (e.g., type I and type III) which are rare (3) and lack clear imaging features, making it difficult to support deep learning models that require large datasets. This not only leads to overfitting during model training but also causes severe class imbalance—common temporal lobe epilepsy data far outnumber extra-temporal cases, biasing model predictions (88). Moreover, in multicenter studies, imaging data originate from different equipment, field strengths, and acquisition protocols, resulting in marked heterogeneity (89). Data annotation relies heavily on expert experience, is highly subjective, and lacks a gold standard; the definition of “MRI-negative” cases remains ambiguous (90), and postoperative pathological confirmation is often insufficient (91), compromising the authenticity and consistency of annotations. Privacy concerns regarding medical data further restrict data sharing and the construction of large-scale repositories. Most studies use limited, non-publicly available databases, and although techniques such as federated learning (92) have been proposed to enable collaborative modeling without sharing raw data, their application remains in early stages.

#### 6.1.2 Model development and training

First, overfitting and insufficient generalizability are common issues. Many models perform excellently in internal validation but show marked performance drops when applied to external independent cohorts; for example, a meta-analysis found that sensitivity and specificity could decline by 8 and 26%, respectively, in external cohorts (93). Second, there is a lack of standardization in hyperparameter selection (e.g., network architecture, learning rate, post-processing thresholds), and the tuning process may inadvertently introduce “data leakage” (94), leading to overly optimistic evaluation results. Third, the complexity of multimodal fusion techniques is high. Effective integration of data from different modalities such as MRI, EEG, and PET requires solving a series of challenges including data alignment, feature extraction (95), and ensemble modeling. Existing pixel-level or feature-level fusion methods often involve trade-offs between spatial resolution and spectral information, and may introduce artifacts or distortions. Additionally, these models are typically computationally expensive, making real-time processing difficult; they are currently mostly confined to offline analysis, which falls short of the demands for immediate clinical decision-making.

#### 6.1.3 Model evaluation and interpretability

The evaluation metrics used across studies are inconsistent: some focus on voxel-wise accuracy (96), while others emphasize lesion-cluster or patient-level diagnostic performance (97), making direct comparisons difficult. More critically, there is a lack of standards for fair comparisons with human experts in real clinical settings experts can integrate multiple non-imaging information sources (e.g., semiology, EEG), whereas models are typically based solely on imaging data; experts usually report only a few highly confident lesions, while models may output numerous suspicious regions (98). In addition, the “black-box” nature of deep learning models (99) renders their decision-making processes opaque, making it difficult for clinicians to understand and trust the outputs, which may pose clinical risks when errors occur.

#### 6.1.4 Clinical translation and regulatory compliance

Integrating model outputs into existing clinical workflows is challenging; clinicians need tools that assist rather than replace their judgment, and translating model results into actionable clinical recommendations remains a major hurdle. At the regulatory level, for AI models to be approved as medical devices (e.g., via FDA or CE certification), they must provide prospective, multicenter, large-sample clinical validation evidence demonstrating their effectiveness and safety in real-world settings a particularly demanding requirement for diseases with small sample sizes. Existing regulatory frameworks also struggle to accommodate adaptive learning models. Currently, most studies remain retrospective, with prospective validation being severely insufficient; the actual impact of these models on clinical decision-making has yet to be fully assessed.

### 6.2 Directions for methodological innovation

#### 6.2.1 Increasing integration of multimodal data

At the data level, the research paradigm is gradually shifting from a predominantly single-modality analytical approach toward deep integration of multimodal data. Through cross-modal registration, feature-level fusion, and advanced co-attention mechanisms (e.g., co-attention, graph attention networks [GAT]), researchers are effectively integrating information from multiple sources including structural MRI, functional MRI, metabolic PET, diffusion DTI, and intracranial/scalp EEG to fully leverage the complementary advantages of different modalities in terms of spatial resolution, temporal dynamics, and physiological specificity, thereby achieving a more comprehensive characterization of the epileptic network.

#### 6.2.2 Increasing prominence of deep learning advantages

Methodologically, although traditional machine learning methods remain widely used, the application of deep learning architectures is rapidly increasing and demonstrating superior performance. Convolutional neural networks (CNNs) and their variants (e.g., 3D-CNN, U-Net) are widely employed for brain image segmentation and lesion detection; recurrent neural networks (RNNs) and temporal convolutional networks (TCNs) effectively process time-series signals such as EEG; and graph neural networks (GNNs) are particularly suited for connectome data analysis. These end-to-end deep learning models circumvent the labor-intensive and subjective manual feature engineering inherent in traditional machine learning, and are capable of automatically learning high-level discriminative features directly from raw data.

#### 6.2.3 Solutions to the small-sample constraint

To address the fundamental challenge of scarce high-quality labeled data in medical applications, a suite of effective technical strategies has been developed and widely adopted. Transfer learning involves pre-training models on large public neuroimaging datasets before fine-tuning them on small epilepsy cohorts, thereby enhancing model performance (100). Data augmentation techniques, such as generative adversarial networks, affine transformation, and noise injection, are utilized to generate synthetic multimodal images, which helps expand the size of training samples and mitigate the risk of overfitting. Federated learning enables multi-center collaborative model training without sharing raw data, allowing the leveraging of aggregated large-scale data while ensuring data privacy (101). Few-shot learning and meta-learning approaches facilitate rapid adaptation to small datasets with limited labeled samples, making them particularly suitable for researching rare epilepsy subtypes (102). Multi-modal attention mechanisms work by weighting informative modalities, which helps suppress noise and improve the efficiency of training on small samples. Additionally, zero-shot and few-shot learning strategies can be actively explored, enabling models to perform effective inference with only minimal or even no target-case data, thereby greatly enhancing applicability in rare epilepsy types or resource-limited settings (103).

#### 6.2.4 Increasing interpretability and clinical translation approaches

As the complexity of artificial intelligence models increases, the emphasis on model interpretability and clinical translation has grown to an unprecedented degree (104). Researchers are now widely employing explainability techniques such as SHAP, Grad-CAM, and class activation mapping (CAM) to clearly reveal the decision-making basis of models, thereby enhancing clinicians’ trust in AI outputs. Concurrently, the field is actively promoting the translation of research findings into clinical practice, with a number of open-source analytical tools (e.g., VEP, MELD) and online prediction platforms having been developed and introduced into clinical trialing. This progress marks a shift in epilepsy AI research from algorithmic innovation toward addressing tangible clinical needs.

## 7 Conclusion

### 7.1 Clinical value and current boundaries

Multimodal AI can integrate anatomical, metabolic, functional, and electrophysiological information to support ASM-response prediction, EZ localization, and surgical-outcome estimation. Its principal value is to reveal complementary patterns that may be missed by any single modality, including subtle abnormalities in selected MRI-negative cases, thereby expanding opportunities for evidence-informed presurgical evaluation and postoperative seizure freedom.

These systems remain adjunctive. Most evidence is retrospective, externally validated models are uncommon, and performance varies with cohort composition and task definition. Accordingly, AI outputs should augment rather than replace multidisciplinary clinical interpretation, and claims of personalized treatment require prospective demonstration of clinical benefit.

### 7.2 Future directions

Looking ahead, the development of multimodal brain imaging-based AI research will advance along several important directions.

At the data level, the challenges of data sharing and privacy protection need to be addressed. Privacy-preserving computing techniques such as federated learning (105) hold promise for enabling multicenter collaborative modeling without the need to centralize raw data. Simultaneously, it is essential to establish industry-recognized standards for key concept definitions, imaging acquisition protocols, and lesion annotation specifications (90). Techniques such as generative adversarial networks (GANs) can be employed for privacy-preserving data augmentation or synthetic data generation to address small-sample and class-imbalance issues. In addition, incorporating external validation from previously unexamined centers is crucial for accurately assessing model performance in real-world clinical scenarios; this approach will enhance model reliability in actual healthcare settings. For instance, the publicly available FCD type II MRI dataset from the University of Bonn Hospital (106) represents a valuable resource for external validation.

Algorithmic and model innovation will constitute another focal area. There is a need to develop or apply more efficient and interpretable multimodal fusion architectures. A straightforward approach to facilitating model comparison is to share code and trained models, enabling others to apply these models to their own data. However, developing ready-to-use code is a labor-intensive task, and applying complex models also requires skills such as programming. Meanwhile, continued improvement in model interpretability is needed to enable clinicians to understand the rationale behind model decisions and build trust. Leveraging foundation models and transfer learning paradigms, models pre-trained on large-scale public natural imaging (107) or healthy brain imaging datasets can be adapted to epilepsy multimodal tasks via transfer learning. This “pre-train then fine-tune” paradigm is expected to significantly reduce the demand for task-specific labeled data and enhance model generalizability. Furthermore, optimizing algorithms to reduce computational complexity is another research direction, requiring the development of real-time or near-real-time processing tools that can be integrated into clinical imaging workstations, thereby shortening analysis time and better aligning with clinical workflows.

Finally, strengthening clinical translation and validation is particularly critical. Future research must transition from retrospective analyses to prospective multicenter clinical validation, rigorously evaluating the efficacy and clinical utility of AI tools in real-world environments. In-depth investigation is also needed into how AI can best collaborate with clinicians (108). Future system designs should not replace physicians, but rather function as “augmented intelligence.” How to effectively present multiple predictive clusters from models to physicians to assist hypothesis generation is a key human-computer interaction challenge that needs to be addressed. In addition, regulatory and ethical frameworks (109) must adapt to the evolution of AI technology. Regulatory agencies should refine dynamic regulatory frameworks (110) that accommodate AI medical devices particularly adaptive learning models establishing safe and effective approval and oversight processes for continuous learning and iterative updates.

In summary, multimodal brain imaging-based AI research is propelling epilepsy diagnosis and treatment from a traditional, expert-experience-dependent paradigm toward a new era of precision medicine characterized by data-driven, multidimensional information integration. Despite the numerous challenges, with continued technological innovation, deepened collaboration, and enhanced awareness of clinical translation, this approach is poised to become an indispensable core component of future epilepsy care systems, ultimately bringing new hope to millions of patients with drug-resistant epilepsy worldwide.
